# Cardiorespiratory Effects of Inverse Ratio Ventilation in Obese Patients During Laparoscopic Surgery: A Systematic Review and Meta-Analysis

**DOI:** 10.3390/jcm14062063

**Published:** 2025-03-18

**Authors:** Michele Carron, Enrico Tamburini, Alessandra Maggiolo, Federico Linassi, Nicolò Sella, Paolo Navalesi

**Affiliations:** 1 Department of Medicine—DIMED, Section of Anesthesiology and Intensive Care, University of Padua, Gallucci V. St. 13, 35121 Padua, Italy; alessandra.maggiolo@studenti.unipd.it (A.M.); paolo.navalesi@unipd.it (P.N.); 2 Institute of Anesthesia and Intensive Care, Padua University Hospital, Giustiniani St. 2, 35128 Padua, Italy; enrico.tamburini@aopd.veneto.it (E.T.); nicolo.sella@aopd.veneto.it (N.S.); 3 Department of Anesthesia and Intensive Care, Ca’ Foncello Treviso Regional Hospital, Hospital Sq. 1, 31100 Treviso, Italy; federicolinassi@gmail.com

**Keywords:** obesity, anesthesia, laparoscopy, ventilation, inverse ratio ventilation, complications

## Abstract

**Background/Objectives:** Managing ventilatory strategies in patients with obesity under general anesthesia presents significant challenges due to obesity-related pathophysiological changes. Inverse ratio ventilation (IRV) has emerged as a potential strategy to optimize respiratory mechanics during laparoscopic surgery in this population. The primary outcomes were changes in respiratory mechanics, including peak inspiratory pressure (P_Peak_), plateau pressure (P_Plat_), mean airway pressure (P_Mean_), and dynamic compliance (C_Dyn_). Secondary outcomes included gas exchange parameters, hemodynamic measures, inflammatory cytokines, and postoperative complications. **Methods:** A systematic review and meta-analysis were conducted, searching PubMed, Scopus, EMBASE, and PMC Central. Only English-language randomized controlled trials (RCTs) evaluating the impact of IRV in adult surgical patients with obesity were included. The quality and certainty of evidence were assessed using the Risk of Bias 2 (RoB 2) tool and the Grades of Recommendation, Assessment, Development, and Evaluation (GRADE) framework, respectively. **Results:** Three RCTs including 172 patients met the inclusion criteria. Compared to conventional ventilation without prolonged inspiratory time or IRV, IRV significantly reduced P_Peak_ (MD [95%CI]: −3.15 [−3.88; −2.42] cmH_2_O, *p* < 0.001) and P_Plat_ (MD [95%CI]: −3.13 [−3.80; −2.47] cmH_2_O, *p* < 0.001) while increasing P_Mean_ (MD [95%CI]: 4.17 [3.11; 5.24] cmH_2_O, *p* < 0.001) and C_Dyn_ (MD [95%CI]: 2.64 [0.95; 4.22] mL/cmH_2_O, *p* = 0.002) during laparoscopy, without significantly affecting gas exchange. IRV significantly reduced mean arterial pressure (MD [95%CI]: −2.93 [−3.95; −1.91] mmHg, *p* < 0.001) and TNF-α levels (MD [95%CI]: −9.65 [−17.89; −1.40] pg/mL, *p* = 0.021). **Conclusions:** IRV optimizes intraoperative respiratory mechanics but has no significant impact on postoperative outcomes, necessitating further research to determine its clinical role.

## 1. Introduction

Ventilation management in patients with obesity under general anesthesia is challenging due to obesity-related physiological changes [1,2]. Obesity reduces lung volumes and impairs respiratory mechanics, with these effects worsening as body mass index (BMI) increases [2]. During laparoscopy, the rise in intra-abdominal pressure further exacerbates hypoventilation and atelectasis, compounding the respiratory impairment associated with obesity [2]. These factors reduce respiratory system compliance, leading to alveolar ventilation/perfusion (V_A_/Q) mismatch, intrapulmonary shunting and arterial hypoxemia [2]. Consequently, maintaining adequate ventilation and oxygenation becomes particularly challenging for anesthesiologists [2].

Optimizing ventilation in patients with obesity is crucial for improving postoperative outcomes [1]. Protective ventilation strategies, including low tidal volumes, individualized positive end-expiratory pressure (PEEP), and low driving pressure, have demonstrated benefits in perioperative care [2]. A network meta-analysis identified volume-controlled ventilation with individualized PEEP and recruitment maneuvers as the optimal strategy, enhancing oxygenation and lung compliance in patients with obesity [3]. However, laparoscopic surgery introduces additional challenges, as carbon dioxide (CO₂) insufflation (pneumoperitoneum) increases intra-abdominal pressure, further impairing lung function [4].

Inverse ratio ventilation (IRV), which prolongs the inspiratory-to-expiratory (I:E) ratio, has emerged as a promising strategy to improve respiratory mechanics in patients with obesity undergoing laparoscopic surgery [5]. Studies suggest that IRV enhances gas exchange and respiratory mechanics in patients with a BMI ≥25 kg/m^2^ [5], as confirmed by a meta-analysis [6]. However, due to the inclusion of patients with and without obesity in these studies [5,6], the evidence remains inconclusive specifically for individuals with obesity, who face increased challenges in lung ventilation during laparoscopy [1,2,4]. While functional residual capacity (FRC) decreases by about 10% in overweight patients, it can drop by up to 33% in those with obesity [7]. Anesthesia induction further compounds these reductions, significantly impacting respiratory mechanics and gas exchange [2], highlighting the need for optimized ventilatory strategies [1].

In light of these complexities and the ongoing debate on the optimal ventilatory strategy for patients with obesity [3], this study aims to provide a comprehensive overview of current evidence and evaluate the effectiveness of IRV in improving intraoperative cardiorespiratory function and postoperative outcomes in patients with obesity undergoing laparoscopic surgery.

## 2. Materials and Methods

The protocol for the meta-analysis was prospectively registered in the PROSPERO database (CRD42024581248, 30 August 2024). The manuscript adheres to Preferred Reporting Items for Systematic Reviews and Meta-Analyses (PRISMA) guidelines [8].

### 2.1. Eligibility Criteria

The criteria for inclusion in this systematic review and meta-analysis were defined according to the PICOS framework as follows:

- Population (P): This study included adult patients (aged ≥18 years) with obesity (BMI ≥30 kg/m^2^) who underwent laparoscopic surgery.
- Intervention (I): Patients received IRV (I:E >1) during mechanical ventilation under general anesthesia.
- Comparison (C): Patients in the control group were ventilated using conventional ventilation without prolonged inspiratory time or IRV.
- Outcomes (O): The primary outcome of interest is the change in respiratory mechanics, including peak inspiratory pressure (P_Peak_), plateau pressure (P_Plat_), mean airway pressure (P_Mean_), PEEP, auto-PEEP, dynamic compliance (C_Dyn_), and static compliance (C_Stat_). The secondary outcomes include the effect on oxygenation (e.g., peripheral arterial oxygenation [SaO_2_], arterial oxygen pressure [PaO_2_], arterial CO_2_ pressure [PaCO_2_], pH, and the PaO_2_/Fraction of inspired oxygen [FiO_2_] ratio [P/F ratio]), hemodynamic parameters (mean arterial pressure [MAP], heart rate [HR]), inflammatory response cytokines (e.g., tumor necrosis factor-alpha [TNF-α], interleukin-1, 6, or 8 [IL-1, IL-6, or IL-8]), intraoperative complications, and postoperative complications. Postoperative complications will be evaluated as a composite of postoperative pulmonary complications (POPCs) [9,10] and non-POPCs, all observed within the postoperative monitoring timeframe. The evaluation was conducted using only pre- and post-pneumoperitoneum measurements. In cases where multiple measurements were available, preference was given to those taken immediately before and after pneumoperitoneum, rather than those taken earlier or later.
- Study Design (S): This review included prospective randomized controlled trials (RCTs) published in English, focusing on adult patients undergoing laparoscopic surgery. Studies were excluded if they were observational, non-clinical, lacked sufficient data or full-text availability, involved obese adults not undergoing laparoscopic surgery, addressed pediatric populations, or were not peer-reviewed.

### 2.2. Search Strategy

The search strategy involved querying electronic databases such as PubMed, Scopus, EMBASE, and PMC Central until 10 January 2025. The focus was on IRV in patients with obesity undergoing laparoscopic surgery. The literature search strategy integrated Medical Subject Headings (MeSHs) and relevant keywords, applying Boolean operators “AND” and “OR” to refine the selection process. The MeSH terms “Obesity” OR “Obesity, Morbid” were combined with “Laparoscopy” OR “Laparoscopes” OR “Laparoscopic Surgery”. To refine and focus the search, the literature search was conducted using the following non-MeSH terms: “Inverse Ratio Ventilation” OR “Inverse Inspiratory to Expiratory Ratio” OR “Prolonged Inspiratory Time” OR “Inspiratory Ratio”. To enhance completeness, the reference lists of selected studies were examined to identify any additional relevant publications. A comprehensive description of the search methodology, including specific keyword combinations and applied filters, is available in Supplementary Material (SM) 1.

### 2.3. Study Selection, Data Extraction, and Data Retrieval

Two independent reviewers (ET, AM) initially screened the titles and abstracts of articles retrieved through the search strategy, excluding non-relevant studies. The full texts of the remaining articles were then evaluated to verify their adherence to the predefined selection criteria. Data extraction was carried out independently by the same reviewers using standardized data collection forms specific to each study. Any discrepancies in study selection, data extraction, or trial assessment were resolved by a third reviewer (MC), who was not involved in the initial search process. Furthermore, two additional authors (FL, NS) conducted a manual review and verification of the selected studies to ensure the accuracy of the extracted data and maintain the integrity of the final dataset.

### 2.4. Quality Assessment and Certainty of Evidence Assessment

Two authors (ET, AM) independently assessed the quality of the included RCTs using the Risk of Bias (RoB) 2 tool [11]. This method evaluates five key domains of potential bias: the randomization process, deviations from intended interventions, missing outcome data, outcome measurement, and the selection of reported results. Each domain is examined through a series of signaling questions designed to detect possible sources of bias. Based on the responses, an algorithm classifies the risk level for each domain as “low”, “high”, or “some concerns” [11]. Any discrepancies in the initial evaluations were addressed through discussion with a third reviewer (MC).

To assess the certainty of evidence for the analyzed outcomes, the Grades of Recommendation, Assessment, Development, and Evaluation (GRADE) framework was employed. This methodology categorizes evidence quality into four levels: high (⊕⊕⊕⊕), moderate (⊕⊕⊕⊖), low (⊕⊕⊖⊖), and very low (⊕⊖⊖⊖) [12]. The starting level for quality of evidence (QoE) derived from RCTs is high, but it may be downgraded due to specific limitations, including risk of bias (e.g., inadequate blinding or allocation concealment), inconsistency (quantified using statistical heterogeneity indices such as I-squared [I²], tau [τ], and tau-squared [τ²]), indirectness (e.g., differences in study populations, interventions, or outcomes relative to the research focus), imprecision (evidenced by wide 95% confidence intervals [CIs] or estimates close to the null effect), and publication bias [12]. For a clearer data interpretation, forest plots were utilized, providing a visual summary of the comparative efficacy and facilitating a more intuitive understanding of the findings.

### 2.5. Statistical Analysis

The meta-analysis was performed using a frequentist approach. For continuous outcomes, the mean difference (MD) and 95% CI were calculated, whereas for binary outcomes, the relative risk (RR) with 95% CI was determined. When studies provided data as the median and interquartile range (IQR), these values were converted into estimated mean and standard deviation (SD) using Wan’s method [13]. Both random-effects and fixed-effects models were considered; however, the random-effects model was preferred due to its ability to better account for heterogeneity across study settings. Dichotomous and continuous variables were analyzed using the inverse variance method, which is particularly suited for handling data variability under random-effects assumptions. In cases where zero events were reported, a 0.5 continuity correction was applied to avoid computational bias. The Mantel–Haenszel method was compared with the inverse variance method, producing comparable results. The inverse-variance weighting employed the DerSimonian and Laird method to account for heterogeneity. A post hoc sensitivity analysis was performed to explore potential sources of heterogeneity, excluding studies with higher BMI ranges, higher PEEP values, and lower I:E ratio values.

Study heterogeneity was assessed using the I² statistic, considering a significance threshold of *p* < 0.1 to indicate its presence. I² values were classified as low (<25%), moderate (25–50%), or high (>50%) [14]. To further characterize heterogeneity, τ was calculated to estimate the standard deviation of true effect sizes across studies, reflecting variation beyond random chance, while τ² was used to quantify between-study variance. In analyses involving a limited number of studies, accurately estimating τ² remained challenging but was crucial for appropriately interpreting heterogeneity levels.

Funnel plots were used for visual inspection to assess the risk of publication bias in the meta-analyses [15]. All analyses were conducted using R software, version 4.3.1 (2023). Consistent with standard statistical practices, all *p*-values were two-tailed, with a significance threshold set at <0.05.

## 3. Results

### 3.1. Paper Selection

**Figure 1:**
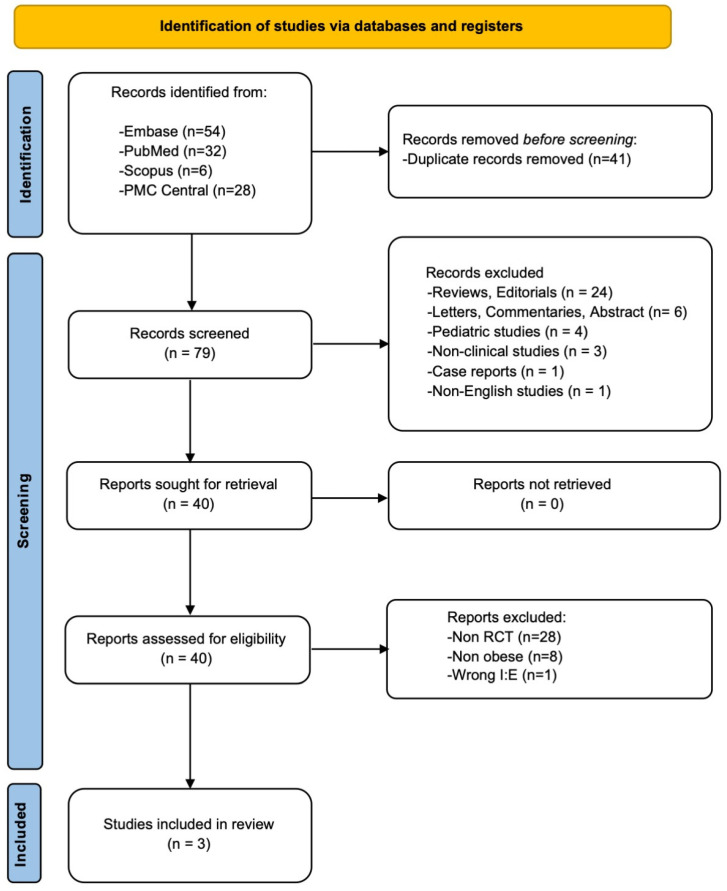
The PRISMA flow diagram of the study selection process.

Out of the 120 reports initially retrieved through the literature search, 117 were excluded as they did not fulfill the inclusion criteria. Consequently, three RCTs comprising a total of 172 patients met the eligibility criteria for inclusion in the meta-analysis [16,17,18]. The study selection process is visually represented in the PRISMA flow diagram (Figure 1).

### 3.2. Study Characteristics

The characteristics of the included RCTs are reported in Table 1 [16,17,18]. Out of the total participants, 86 patients were allocated to the treatment, while 86 were allocated to the control [16,17,18].

### 3.3. Risk of Bias Assessment

**Figure 2:**
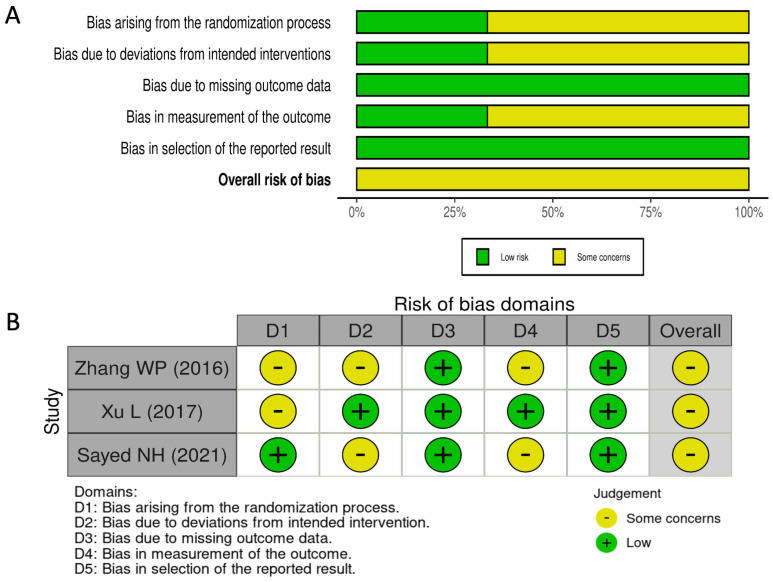
Summary plot (**A**) and traffic-light plot (**B**) of RoB 2 assessment for the included RCTs [16,17,18].

The RoB 2 evaluation of the included RCTs suggests that these studies exhibit either low or unclear risk of bias. The distribution of bias assessments across different domains is illustrated in Figure 2, while SM2 provides a detailed breakdown of domain-specific judgments for each study. Although all studies were reported as randomized, some lacked clarity regarding the randomization procedure, allocation concealment, or masking strategies [16,17]. Furthermore, certain studies did not specify the blinding method applied to operators and participants [16,18]. However, all included studies presented outcome data aligned with their predefined endpoints [16,18]. Only one study explicitly mentioned that the outcome assessor was blinded to the intervention received by participants [17], while in the others, this aspect was not clearly defined [16,18]. Outcome measurements and analyses followed a pre-specified plan to minimize the potential for biased result selection [16,17,18]. Nevertheless, some concerns remain regarding the overall risk of reporting bias.

Forest plots illustrating the estimated treatment effects and confidence intervals are available in SM3, while Table 2 and Table 3 summarize the results for all analyzed outcomes. Additionally, SM4 includes funnel plots used to assess potential publication bias in the meta-analyses.

### 3.4. Primary Endpoint

Compared to the control, IRV reduced P_Peak_ (MD [95%CI]: −2.23 [−3.60;−0.86] cmH_2_O, *p* = 0.001, moderate QoE) and P_Plat_ (MD [95%CI]: −1.82 [−2.80;−0.84] cmH_2_O, *p* < 0.001, moderate QoE), and increased P_Mean_ (MD [95%CI]: 2.23 [1.94; 2.51] cmH_2_O, *p* < 0.001, high QoE) [16,18] and C_Dyn_ (MD [95%CI]: 2.03 [0.81; 3.24] mL/cmH_2_O, *p* = 0.001, moderate QoE) before pneumoperitoneum (Table 2) [16,17,18]. Similarly, during pneumoperitoneum, IRV reduced P_Peak_ (MD [95%CI]: −3.15 [−3.88; −2.42] cmH_2_O, *p* < 0.001, high QoE) and P_Plat_ (MD [95%CI]: −3.13 [−3.80; −2.47] cmH_2_O, *p* < 0.001, high QoE), and increased P_Mean_ (MD [95%CI]: 4.17 [3.11; 5.24] cmH_2_O, *p* < 0.001, moderate QoE) [16,18] and C_Dyn_ (MD [95%CI]: 2.64 [0.95; 4.32] mL/cmH_2_O, *p* = 0.002, moderate QoE) (Table 3) [16,17,18]. At the sub-analysis, excluding the study with patients in the Trendelenburg position [16], IRV showed a greater benefit in patients placed in the reverse Trendelenburg position increasing C_Dyn_ before (MD [95% CI]: 2.46 [0.15; 4.76] mL/cmH_2_O, *p* = 0.036, I^2^ [95% CI]: 58.4% [0.0%; 90.2%], moderate QoE) and during pneumoperitoneum (MD [95% CI]: 3.16 [1.01; 5.31] mL/cmH_2_O, *p* = 0.004, I² [95% CI]: 75.5% [0.0%; 94.5%], moderate QoE) [17,18]. Although PEEP and C_Stat_ were initially considered as part of the primary outcome, these variables were not available in the studies we reviewed, and therefore were not included in the final analysis.

A post hoc sensitivity analysis showed that excluding the study with higher BMI [18] reduced C_Dyn_ before pneumoperitoneum (MD [95%CI]: 1.58 [0.43;2.74] mL/cmH₂O, *p* = 0.006, I² [95%CI]: 21.5% [0.0%; 99.9%]; moderate QoE). This suggests that IRV improves dynamic compliance in patients with obesity, with a more pronounced effect in those with severe obesity before pneumoperitoneum. However, data on post-pneumoperitoneum effects were not available, preventing further conclusions on this phase. Excluding the study with PEEP 5 cmH₂O [17] reduced heterogeneity (I^2^ = 0%) without affecting P_Peak_ or P_Plat_, suggesting that IRV’s effect on airway pressures is independent of PEEP levels within the studied range. Removing the study with I:E = 1.5:1 [17] resulted in a higher C_Dyn_ before pneumoperitoneum (MD [95%CI]: 2.57 [0.46; 4.68], *p* = 0.016, I^2^ [95%CI]: 47.3% [0.0%; 99.9%], moderate QoE) compared to the total dataset (MD [95%CI]: 2.03 [0.81; 3.24], *p* = 0.001, I^2^ [95%CI]: 21.5% [0.0%; 91.8%], moderate QoE). However, during pneumoperitoneum, C_Dyn_ was lower after excluding this study (MD [95%CI]: 1.77 [0.66; 2.87], *p* = 0.001, I² [95%CI]: 0.0% [14%; 99%] moderate QoE) compared to the total dataset (MD [95%CI]: 2.64 [0.95; 4.32], *p* = 0.002, I² [95%CI]: 74.2% [14%; 92.3%], moderate QoE). This suggests that a shorter inspiratory phase (e.g., I:E = 1.5:1) may have helped maintain compliance under normal conditions but was less effective in preserving compliance under increased intra-abdominal pressure.

### 3.5. Secondary Endpoints

#### 3.5.1. Gas Exchange

Compared to the control, IRV did not impact PaO_2_ (MD [95%CI]: 2.49 [−6.32; 11.31] mmHg, *p* = 0.579, low QoE), PaCO_2_ (MD [95%CI]: −0.09 [−1.75;1.56] mmHg, *p* = 0.911, low QoE), and pH (MD [95%CI]: −0.001 [−0.02; 0.01], *p* = 899, low QoE) before pneumoperitoneum (Table 2) [16,18]. During pneumoperitoneum, similar results were observed with no impact on PaO_2_ (MD [95%CI]: 20.9 [−1.55; 43.48] mmHg, *p* = 0.068, low QoE), and PaCO_2_ (MD [95%CI]: −0.96 [−6.15; 4.22] mmHg, *p* = 0.716, low QoE), but there was an effect on pH (MD [95%CI]: −0.03 [−0.05;−0.01], *p* < 0.001, moderate QoE) (Table 3) [16,18].

#### 3.5.2. Hemodynamics

Compared to the control, IRV did not impact PAM (MD [95%CI]: −1.11 [−2.41; 0.18] mmHg, *p* < 0.092, moderate QoE) and HR (MD [95%CI]: −0.45 [−1.60; 0.69] beats/min, *p* = 0.440, moderate QoE) before pneumoperitoneum (Table 2) [16,18]. However, during pneumoperitoneum, IRV impacted PAM (MD [95%CI]: −2.93 [−3.95;−1.91] mmHg, *p* < 0.001, moderate QoE), but not HR (MD [95%CI]: −1.08 [−3.19; 1.01] beats/min, *p* = 0.310, low QoE) (Table 3) [16,18].

#### 3.5.3. Inflammatory Cytokines

Compared to the control, IRV did not impact TNF−α (MD [95%CI]: −1.76 [−10.74;7.22] pg/mL, *p* = 0.700, low QoE) before pneumoperitoneum (Table 2), but it did have an impact during pneumoperitoneum (MD [95%CI]: −9.65 [−17.89;−1.40] pg/mL, *p* = 0.020, moderate QoE) (Table 3) [16,17].

#### 3.5.4. Postoperative Complications

Compared to the control, IRV did not impact postoperative complications (RR [95%CI]: 0.24 [0.01; 6.44], *p* = 0.4022, I^2^ [95%CI]: 94.3% [82.1%; 98.2%], low QoE) [16,17,18].

## 4. Discussion

This study demonstrated that IRV significantly improved respiratory mechanics in patients with obesity undergoing laparoscopic surgery but had a minimal impact on gas exchange and hemodynamic stability. It was also associated with reduced inflammatory cytokine levels during pneumoperitoneum but did not significantly impact postoperative complications. These findings suggest that while IRV enhances respiratory mechanics in patients with obesity undergoing laparoscopic surgery, its overall clinical benefits remain uncertain.

Patients with obesity undergoing laparoscopic surgery typically exhibit reduced FRC, further complicated by the positioning and pneumoperitoneum [5]. Low lung compliance leads to increased atelectasis and heterogeneous ventilation [1,2], predisposing patients to V_A_/Q mismatch and worsening respiratory impairment [5]. Consequently, optimizing ventilation in this population remains challenging [1]. IRV, which aims to increase FRC, has been considered a promising strategy in patients with obesity [5,16,17,18]. By altering the I:E ratio, IRV prolongs the inspiratory phase and reduces inspiratory flow, thereby lowering P_Peak_ and P_Plateau_ pressures. Extending the inspiratory phase also allows more time for gas to enter the alveoli, improving the recruitment of under-ventilated regions and enhancing P_Mean_ and C_Dyn_ in obese patients [17]. However, the auto-PEEP generated by a shortened expiratory phase may lead to alveolar overdistension or relative hyperinflation of already well-ventilated units (V_A_/Q >1) and can elevate intrathoracic pressure, potentially impairing pulmonary and systemic circulation as well as gas exchange efficiency, especially in patients with reduced lung compliance [5,16]. A study demonstrated that increasing the I:E ratio from 2:1 to 4:1 led to a progressive rise in auto-PEEP from 0 to 3.5 cm H_2_O and a decrease in MAP from 98.2 mmHg to 83.2 mmHg [5]. Although our analysis did not show a consistent effect of IRV on oxygenation, evidence suggests that IRV can increase FRC, thereby reducing physiological and shunt dead spaces, and improving oxygenation in a mixed population of patients with and without obesity [5]. However, these benefits may be offset by IRV-induced auto-PEEP and V_A_/Q mismatch, which could counteract its positive effects [5]. It has been shown that as the I:E ratio increases, both alveolar and shunt dead spaces also increase, suggesting worsening V_A_/Q mismatch, which is characteristic of hyperinflated, poorly ventilated alveoli [5]. Therefore, while IRV improves certain respiratory mechanics, an I:E ratio higher than 2:1 does not appear to confer additional benefits in obese patients undergoing laparoscopic surgery [5]. Trendelenburg positioning significantly affects respiratory mechanics by increasing P_Peak_, P_Plat_, P_Mean_ while decreasing C_Dyn_ due to a reduction in FRC. In contrast, reverse Trendelenburg positioning does not provide a clinically meaningful improvement in respiratory mechanics [4,19].

IRV should be integrated into a lung-protective strategy, an approach proven to reduce POPCs [20]. Its application should be guided by respiratory mechanics and oxygenation to ensure appropriate use [20]. Rather than being applied to all patients with obesity, IRV should be prioritized in those with severe obesity or significant respiratory mechanical impairment, where it can be tailored to optimize lung mechanics and maintain alveolar recruitment. This is particularly relevant during laparoscopic or robotic-assisted surgery, where prolonged pneumoperitoneum and steep Trendelenburg positioning further exacerbate respiratory impairment [5,18]. In this context, ventilatory settings—including the I:E ratio—should be dynamically adjusted based on surgical phases, as compliance varies during pneumoperitoneum. A tailored approach may optimize respiratory mechanics and minimize the limitations of fixed ventilation settings.

Beyond cardiac factors such as preload, afterload, cardiac contractility, heart rate, and myocardial compliance, as well as volume status, intra-abdominal pressure and patient positioning should also be carefully considered as contributing factors impacting MAP during pneumoperitoneum [4]. Hypercarbia, another contributing factor, is typically avoided during laparoscopy through appropriate ventilatory adjustments [4]. Increased intra-abdominal pressure is the main driver of hemodynamic changes during laparoscopy, typically raising MAP due to increased systemic vascular resistance [4]. However, positioning also plays a significant role [4]. The Trendelenburg position increases venous return and cardiac filling pressures, thereby raising preload and MAP. However, it may paradoxically reduce cardiac output by increasing afterload due to elevated systemic vascular resistance [4]. Conversely, the reverse Trendelenburg position can lower MAP and cause hypotension due to reduced preload and venous pooling, worsened by femoral venous compression from increased intra-abdominal pressure [4]. In studies evaluating IRV in the reverse Trendelenburg position, both the IRV and control groups experienced MAP reductions, likely due to decreased venous return, which minimized IRV’s impact on MAP [17,18]. Conversely, studies conducted in the Trendelenburg position showed significant MAP differences, likely reflecting IRV’s primary effect despite enhanced venous return [5,16]. Our meta-analysis confirmed that IRV significantly affected MAP only during pneumoperitoneum, emphasizing the importance of considering patient positioning and its interaction with ventilatory strategies when assessing hemodynamic outcomes during laparoscopic surgery in patients with obesity.

Studies suggest that IRV reduces the release of inflammatory cytokines, such as TNF-α, which play a crucial role in lung injury associated with conventional ventilation [16,17]. IRV has been reported to lower levels of TNF-α, IL-6, and IL-8 during pneumoperitoneum, potentially attenuating inflammation and reducing ventilator-induced lung injury risk [16,17]. Our meta-analysis supports these findings, demonstrating a significant TNF-α reduction with IRV during surgery, highlighting its dual benefits in improving respiratory mechanics and mitigating inflammation in patients with obesity undergoing laparoscopic procedures. Additionally, this benefit has been observed to peak at 24 h post-surgery and extend beyond 48 h after surgery [17]. Although the clinical impact of TNF-α reduction remains uncertain, lower TNF-α levels have been associated with reduced ventilator-induced lung injury [21]. Given its role in inflammation and alveolar damage, the observed TNF-α reduction with IRV may indicate a protective effect [21], though further studies are needed to confirm its clinical significance.

Despite the potential benefits of IRV in improving respiratory mechanics and reducing inflammation, our meta-analysis did not show a significant difference in postoperative complications, particularly POPCs, compared to standard ventilation. This aligns with evidence suggesting that while lung-protective strategies (e.g., low tidal volume with PEEP) reduce pulmonary complications by 63% and atelectasis by 61% [22], the impact of specific ventilation strategies, including IRV, remains uncertain. Furthermore, Neto et al. demonstrated that driving pressure, rather than tidal volume or PEEP alone, is a key determinant of POPCs; each unit increases significantly raising the risk by 16% [23]. Thus, while IRV optimizes certain respiratory parameters, its clinical benefit depends on maintaining a P_Plat_ <30 cmH_2_O and a driving pressure <13–15 cmH_2_O, thresholds linked to a lower risk of ventilator-induced lung injury and POPCs [22,23]. If IRV increases driving pressure, it may counteract its potential advantages by raising the risk of lung injury.

### Strengths and Limitations of the Study

This meta-analysis is the first to explore the impact of IRV in patients with obesity undergoing laparoscopic surgery, emphasizing its potential to optimize ventilatory strategies and laying the foundation for further research on respiratory mechanics, oxygenation, and postoperative outcomes. The study is grounded in a comprehensive literature review, reducing the risk of overlooking key studies and ensuring a robust dataset. By focusing exclusively on RCTs in adults with obesity, the findings are highly relevant and directly applicable to this patient group. Furthermore, the integration of the GRADE assessment strengthens the reliability of the conclusions, offering a clear and transparent evaluation of evidence certainty.

Several limitations are acknowledged in this study. First, the inclusion of only three single-center RCTs with a small sample size limits statistical power and generalizability, particularly for postoperative outcomes. Caution is required when interpreting our findings, and larger, multicenter trials are necessary to confirm IRV’s effects in patients with obesity undergoing laparoscopic surgery. The wide confidence intervals in some effect estimates suggest potential imprecision, likely due to the small number of included studies. While leave-one-out sensitivity analyses were considered, subgroup analyses provided more relevant insights into heterogeneity and supported the robustness of our findings regarding IRV’s effects on respiratory mechanics. However, its impact on postoperative outcomes remains uncertain, and its broader clinical utility requires further validation.

Second, potential sources of bias were identified. The RoB 2 assessment revealed concerns regarding allocation concealment and blinding in some studies, which may introduce performance and detection bias. However, as key respiratory outcomes (e.g., P_Peak_, P_Plat_, C_Dyn_) are objective physiological measures, their impact is expected to be minimal.

Third, variations in ventilatory strategies across studies may have influenced baseline conditions before IRV evaluation [16,17,18]. Differences in I:E ratios remain an open question, as a formal meta-regression was not feasible. However, sensitivity analyses addressed these variations, and further research is needed to define the optimal IRV parameters.

Additionally, key respiratory parameters were not fully assessed, limiting a comprehensive evaluation of IRV’s effect on lung mechanics. Static compliance—a key indicator of lung stiffness, particularly in patients suffering from obesity with altered lung mechanics—was not reported in the included studies. Additionally, auto-PEEP was either inaccurately measured due to instrumentation limitations [16] or not assessed in some studies [17,18]. Consequently, conclusions regarding the impact of IRV on auto-PEEP are based on studies involving mixed populations of patients with and without obesity [5].

Fourth, the exclusion of non-RCTs, conference proceedings, and grey literature may have increased the risk of publication bias. Although the funnel plot did not indicate strong bias, the small number of studies and variability in outcomes suggest that selective reporting cannot be entirely excluded.

Finally, the analysis of inflammatory cytokines was limited to TNF-α. Although some studies evaluated IL-6 and IL-8 [16] and surfactant protein A, a biomarker of lung injury [17], differences in measurement methods and timing prevented a reliable meta-analysis. Specifically, TNF-α was measured in serum at the end of surgery in one study [17] and in bronchoalveolar lavage (BAL) fluid during pneumoperitoneum in another [16], introducing variability. Given their role in lung inflammation, future studies should further investigate these markers and standardize measurement protocols to assess IRV’s broader impact.

## 5. Conclusions

While IRV shows promise in improving respiratory mechanics, its effects on oxygenation and POPCs remain inconclusive. The interaction between ventilation strategies—including lung-protective approaches—and driving pressure appears to be crucial, warranting further research to clarify IRV’s impact on postoperative outcomes. Additionally, the observed reduction in inflammatory cytokine levels, specifically TNF-α, suggests a potential modulatory effect on inflammation during laparoscopic surgery. However, its clinical relevance and long-term benefits remain to be fully elucidated.

## Figures and Tables

**Table 1:** Characteristics of studies considered for review and meta-analysis.

Author Study (Year)	Country	Surgery	Population (BMI) Intervention/Control	Intervention Group I:E	Intervention Group Ventilatory Setting	Control Group I:E	Control Group Ventilatory Setting	Position (Pneumo-Pressure)	Primary Endpoint
Zhang WP et al. (2016) [16]	China	Gynecological LPS surgery, duration >1 h	Obese (30 < BMI < 35 kg/m^2^) 30/30	2:1	VCV TV 8 mL/kg _(ABW)_ PEEP 0 cmH_2_O RR 12 breath/min FiO_2_: 100%	1:2	VCV TV 8 mL/kg _(ABW)_ PEEP 0 cmH_2_O RR 12 breath/min FiO_2_: 100%	30° Trendelemburg (15 mmHg)	Changes in PaO_2_
Xu L et al. (2017) [17]	China	Gynecological LPS surgery, duration >2 h	Obese (BMI > 30 kg/m^2^) 30/30	1.5:1	PCIRV P_Insp_ = P_Peak_ with VCV PEEP 5 cmH_2_O RR to PetCO_2_ < 50 mmHg FiO_2_: 21–89%	1:2	VCV TV 8 mL/kg _(IBW)_ PEEP 5 cmH_2_O RR to PetCO_2_ < 50 mmHg FiO_2_: 21–89%	30° Reverse Trendelemburg (14–15 mmHg)	Changes in VT dynamic CRS, ΔP, PaO_2_/FiO_2_, PaCO_2_, SP-A, TNF-α.
Sayed NH et al. (2021) [18]	Egypt	LSG	Obese (35 < BMI < 50 kg/m^2^) 26/26	2:1	VCV TV 8 mL/kg _(IBW)_ PEEP 0 cmH_2_O RR 12 breath/min FiO_2_: 60%	1:2	VCV TV 8 mL/kg _(IBW)_ PEEP 0 cmH_2_O RR 12 breath/min FiO_2_: 60%	30° Reverse Trendelemburg (15 mmHg)	Changes in PaO_2_

ABW: Actual Body Weight; BMI: Body Mass Index; CRS: Respiratory System Compliance; I:E: Inspiratory-to-Expiratory ratio; IBW: Ideal Body Weight; LPS: Laparoscopic; LSG: Laparoscopic Sleeve Gastrectomy; PCIRV: Pressure-Controlled Inverse Ratio Ventilation; PEEP: Positive End-Expiratory Pressure; P_Insp_: Inspiratory Pressure; P_Peak_: Peak Airway Pressure; RR: Respiratory Rate; TV: Tidal Volume; VCV: Volume-Controlled Ventilation.

**Table 2:** Effects of IRV on respiratory and hemodynamic variables before CO_2_ pneumoperitoneum.

Respiratory Mechanics								
Variable	MD	95%CI	*p*-Value	τ^2^	τ	I^2^ [95%CI]	*p* = at Q test	QoE
P_Peak_ (cmH_2_O)	−2.23	[−3.60;−0.86]	0.001	0.821	0.906	83.8% [33.1%; 96.1%]	0.012	⊕⊕⊕⊖ Moderate ^§^
P_Plat_ (cmH_2_O)	−1.82	[−2.80;−0.84]	<0.001	0.353	0.594	70.6% [0.0%; 93.4%]	0.065	⊕⊕⊕⊖ Moderate ^§^
P_Mean_ (cmH_2_O)	2.23	[1.94; 2.51]	<0.001	0.0	0.0	0.0% [0.0%; 0.0%]	0.613	⊕⊕⊕⊕ High
C_Dyn_ (mL/cmH_2_O)	2.03	[0.81; 3.24]	0.001	0.256	0.506	21.5% [0.0%; 91.8%]	0.279	⊕⊕⊕⊖ Moderate ^§^
**Gas exchange**								
**Variable**	**MD**	**95%CI**	***p*-Value**	**τ^2^**	**τ**	**I^2^ [95%CI]**	***p* = at Q test**	**QoE**
PaO_2_ (mmHg)	2.49	[−6.32; 11.31]	0.579	36.391	6.032	89.9% [62.6%;97.2%]	0.001	⊕⊕⊖⊖ Low ^§#^
PaCO_2_ (mmHg)	−0.09	[−1.75;1.56]	0.911	0.881	0.938	61% [0.0%;90.9%]	0.109	⊕⊕⊖⊖ Low ^§#^
pH	−0.001	[−0.02;0.01]	0.899	<0.001	0.012	79.2% [9.8%;95.2%]	0.028	⊕⊕⊖⊖ Low ^§#^
**Hemodynamics**								
**Variable**	**MD**	**95%CI**	***p*-value**	**τ^2^**	**τ**	**I^2^ [95%CI]**	***p* = at Q test**	**QoE**
PAM (mmHg)	−1.11	[−2.41; 0.18]	0.092	0.0	0.0	0.0% [0.0%; 0.0%]	0.696	⊕⊕⊕⊖ Moderate ^#^
HR (beats/min)	−0.45	[−1.60; 0.69]	0.440	0.0	0.0	0.0% [0.0%; 0.0%]	0.464	⊕⊕⊕⊖ Moderate ^#^
**Inflammatory cytokines**								
**Variable**	**MD**	**95%CI**	***p*-Value**	**τ^2^**	**τ**	**I^2^ [95%CI]**	***p* = at Q test**	**QoE**
TNF-α (pg/mL)	−1.76	[−10.74; 7.22]	0.700	37.477	6.121	88.6% [56.5%; 97%]	0.003	⊕⊕⊖⊖ Low ^§#^

P_Peak_: Peak inspiratory pressure; P_Plat_: Plateau pressure; P_Mean_: Mean airway pressure: C_Dyn_: Dynamic compliance; PaO_2_: Arterial oxygen pressure; PaCO_2_: Arterial carbon dioxide pressure; PAM: Mean arterial pressure; HR: Heart rate; TNF-α: Tumor necrosis factor-alpha; MD: Mean Difference; 95%CI: 95% Confidence Interval; τ²: Between-study variance in random-effects meta-analysis; τ: Standard deviation estimate of effect sizes in random-effects meta-analysis; I²: Measures percentage variation across studies due to heterogeneity; Q test: Cochran’s Q test assesses heterogeneity among study results; QoE: Quality of Evidence. ^§^ Downgraded one level for inconsistency, reflecting heterogeneity in effect estimates across trials. Although a low I² value generally suggests minimal heterogeneity, the QoE remains uncertain when associated with a broad I² confidence interval, indicating potential undetected heterogeneity. In such instances, the QoE for the outcome was prudently downgraded by one level [12]. ^#^ Downgraded one level for imprecision, particularly when 95% confidence intervals were wide and either included or were close to the null effect [12]. High QoE (⊕⊕⊕⊕): The authors are very confident that the true effect is close to the estimated effect. Moderate QoE (⊕⊕⊕⊖): The authors have moderate confidence in the estimate. The true effect is likely close to the estimate but may differ significantly. Low QoE (⊕⊕⊖⊖): The authors’ confidence in the estimate is limited, and the true effect could substantially deviate from the reported value [12].

**Table 3:** Effects of IRV on respiratory and hemodynamic variables during CO_2_ pneumoperitoneum.

**Respiratory Mechanics**								
**Variable**	**MD**	**95%CI**	***p*-Value**	**τ^2^**	**τ**	**I^2^ [95%CI]**	***p* = at Q test**	**QoE**
P_Peak_ (cmH_2_O)	−3.15	[−3.88; −2.42]	<0.001	0.0	0.0	0.0% [0.0%; 0.0%]	0.537	⊕⊕⊕⊕ High
P_Plat_ (cmH_2_O)	−3.13	[−3.80; −2.47]	<0.001	0.0	0.0	0.0% [0.0%; 0.0%]	0.886	⊕⊕⊕⊕ High
P_Mean_ (cmH_2_O)	4.17	[3.11; 5.24]	<0.001	0.489	0.699	80.9% [18.5%; 95.5%]	0.022	⊕⊕⊕⊖ Moderate ^§^
C_Dyn_ (mL/cmH_2_O)	2.64	[0.95; 4.32]	0.002	1.64	1.28	74.2% [14%; 92.3%]	0.020	⊕⊕⊕⊖ Moderate ^§^
**Gas exchange**								
**Variable**	**MD**	**95%CI**	***p*-Value**	**τ^2^**	**τ**	**I^2^ [95%CI]**	***p* = at Q test**	**QoE**
PaO_2_ (mmHg)	20.96	[−1.55; 43.48]	0.068	203.794	14.275	71.4% [0.0%; 93.6%]	0.061	⊕⊕⊖⊖ Low ^§#^
PaCO_2_ (mmHg)	−0.96	[−6.15; 4.22]	0.716	12.893	3.590	91.8% [71.5%; 97.6%]	0.000	⊕⊕⊖⊖ Low ^§#^
pH	−0.03	[−0.05; −0.01]	<0.001	<0.001	0.005	12.5% [0.0%; 99.9%]	0.285	⊕⊕⊕⊖ Moderate ^§^
**Hemodynamics**								
**Variable**	**MD**	**95%CI**	***p*-Value**	**τ^2^**	**τ**	**I^2^ [95%CI]**	***p* = at Q test**	**QoE**
PAM (mmHg)	−2.93	[−3.95; −1.91]	<0.001	0.030	0.175	1.9% [0.0%; 99.9%]	0.312	⊕⊕⊕⊖ Moderate ^§^
HR (beats/min)	−1.08	[−3.19; 1.01]	0.310	0.747	0.864	23.9% [0.0%;99.9%]	0.251	⊕⊕⊖⊖ Low ^§#^
**Inflammatory cytokines**								
**Variable**	**MD**	**95%CI**	***p*-Value**	**τ^2^**	**τ**	**I^2^ [95%CI]**	**p= at Q test**	**QoE**
TNF-α (pg/mL)	−9.65	[−17.89;−1.40]	0.021	29.510	5.432	81.7% [22.4%;95.7%]	0.019	⊕⊕⊕⊖ Moderate ^§^

P_Peak_: Peak inspiratory pressure; P_Plat_: Plateau pressure; P_Mean_: Mean airway pressure: C_Dyn_: Dynamic compliance; PaO_2_: Arterial oxygen pressure; PaCO_2_: Arterial carbon dioxide pressure; PAM: Mean arterial pressure; HR: Heart rate; TNF-α: Tumor necrosis factor-alpha; MD: Mean Difference; 95%CI: 95% Confidence Interval; τ^2^: Between-study variance in random-effects meta-analysis; τ: Standard deviation estimate of effect sizes in random-effects meta-analysis; I²: Measures percentage variation across studies due to heterogeneity; Q test: Cochran’s Q test assesses heterogeneity among study results; QoE: Quality of Evidence. ^§^ Downgraded one level for inconsistency, reflecting heterogeneity in effect estimates across trials. Although a low I^2^ value generally suggests minimal heterogeneity, the QoE remains uncertain when associated with a broad I^2^ confidence interval, indicating potential undetected heterogeneity. In such instances, the QoE for the outcome was prudently downgraded by one level [12]. ^#^ Downgraded one level for imprecision, particularly when 95% confidence intervals were wide and either included or were close to the null effect [12]. High QoE (⊕⊕⊕⊕): The authors are very confident that the true effect is close to the estimated effect. Moderate QoE (⊕⊕⊕⊖): The authors have moderate confidence in the estimate. The true effect is likely close to the estimate but may differ significantly. Low QoE (⊕⊕⊖⊖): The authors’ confidence in the estimate is limited, and the true effect could substantially deviate from the reported value [12].

## Data Availability

The data used in this meta-analysis were extracted from publicly available studies. The extracted dataset is available from the corresponding author upon reasonable request.

## Supplementary Materials

The following supporting information can be downloaded at: https://www.mdpi.com/article/10.3390/jcm14062063/s1. PRISMA 2020 checklist; Supplementary Material (SM) 1: Search strategy; SM2: Reasons for the risk of bias judgments; SM3: Forest plots; SM4: Funnel plots.
