# Supplementary material for: Cardiorespiratory Effects of Inverse Ratio Ventilation in Obese Patients During Laparoscopic Surgery: A Systematic Review and Meta-Analysis
Source: J Clin Med. 2025 Mar 18;14(6):2063. doi: 10.3390/jcm14062063 (PMC11943165; doi:10.3390/jcm14062063)

Search strategy

A comprehensive literature search was conducted to identify relevant studies for inclusion in this systematic review and meta-analysis. The databases queried included PubMed, Scopus, EMBASE and PMC central.

**PubMed**	**((((("inverse ratio ventilation") OR ("prolonged inspiratory time")) OR ("inspiratory ratio")) OR ("Inverse Inspiratory to Expiratory Ratio")) OR ("Inverse I Ratio")) AND (((obesity[MeSH Terms]) OR (morbid obesity[MeSH Terms])) OR (obes*))) AND ((((laparoscopy[MeSH Terms]) OR (laparoscopes[MeSH Terms])) OR (laparoscopic surgery[MeSH Terms])) OR (laparoscop*))** ("inverse ratio ventilation"[All Fields] OR "prolonged inspiratory time"[All Fields] OR "inspiratory ratio"[All Fields] OR "Inverse Inspiratory to Expiratory Ratio"[All Fields] OR (("inverse"[All Fields] OR "inversed"[All Fields] OR "inversely"[All Fields] OR "inverses"[All Fields]) AND ("ratio"[All Fields] OR "ratio s"[All Fields] OR "ratioes"[All Fields] OR "ratios"[All Fields]))) AND ("obesity"[MeSH Terms] OR "obesity, morbid"[MeSH Terms] OR "obes*"[All Fields]) AND ("laparoscopy"[MeSH Terms] OR "laparoscopes"[MeSH Terms] OR "laparoscopy"[MeSH Terms] OR "laparoscop*"[All Fields])
**Scopus**	((TITLE-ABS-KEY (laparoscopy) OR TITLE-ABS-KEY (laparoscopic AND surgery) OR TITLE-ABS-KEY (laparoscopes) OR TITLE-ABS-KEY (laparoscop*))) AND ((TITLE-ABS-KEY (obes*) OR TITLE-ABS-KEY (obesity) OR TITLE-ABS-KEY (morbid AND obesity))) AND ((TITLE-ABS-KEY ("inverse ratio ventilation") OR TITLE-ABS-KEY ("prolonged inspiratory time") OR TITLE-ABS-KEY ("inspiratory ratio") OR TITLE-ABS-KEY ("inverse I ratio")))

**Embase**	1	("inverse ratio ventilation" or "prolonged inspiratory time" or "inspiratory ratio" or "Inverse Inspiratory to Expiratory Ratio" or "Inverse I Ratio").mp. [mp=tx, bt, ti, ab, ct, kw, ot, fx, sh, hw, bo, tn, dm, mf, dv, kf, dq, nm, ox, px, rx, ui, sy, ux, mx, rv]
	2	(obesity or obes* or morbid obesity).mp. [mp=tx, bt, ti, ab, ct, kw, ot, fx, sh, hw, bo, tn, dm, mf, dv, kf, dq, nm, ox, px, rx, ui, sy, ux, mx, rv]
	3	(laparoscopy or laparoscopes or laparoscopic surgery or laparoscop*).mp. [mp=tx, bt, ti, ab, ct, kw, ot, fx, sh, hw, bo, tn, dm, mf, dv, kf, dq, nm, ox, px, rx, ui, sy, ux, mx, rv]
	4	1 and 2 and 3
**PMC Central**	Search ((((((((laparoscopy[MeSH Terms]) OR laparoscopes[MeSH Terms]) OR laparoscopic surgery[MeSH Terms]) OR laparoscop*)) AND (((obesity[MeSH Terms]) OR morbid obesity[MeSH Terms]) OR obes*)) AND ((((("inverse ratio ventilation") OR "prolonged inspiratory time") OR "inspiratory ratio") OR "Inverse Inspiratory to Expiratory Ratio") OR "Inverse I Ratio"))))	

Risk of bias assessment based on revised Cochrane risk-of-bias tool for randomized trials (RoB 2).

Below, the reasons for the RoB 2 assessment of each Randomized Controlled Trial (RCT) included in the meta-analysis are presented. The RoB 2 tool evaluates five key domains of bias risk: the randomization process, deviations from intended interventions, missing outcome data, measurement of the outcome, selection of the reported result, and an overall RoB 2 assessment. Within each domain, a series of questions (referred to as 'signaling questions') are posed to identify potential sources of bias. Based on the answers to these signaling questions, an assessment of bias for each domain is proposed, ranging from 'low' or 'high' risk to 'some concerns'. The overall RoB 2 assessment is then derived from these domain-specific evaluations.

Zhang WP (2016)

Entry	Judgment	Description
**Randomization process**	*Some concerns*	Block randomization using a computer-generated random allocation sequence reported. Allocation sequence concealed not described. No apparent imbalances.
**Deviations from intended interventions**	*Some concerns*	Not specified whether anesthesiologist delivering the interventions and/or patients and/or outcome assessor are not aware of the participants' assigned interventions. No apparent imbalances.
**Missing outcome data**	*Low risk*	Data about outcomes available for all participants. Result not biased by missing outcome data.
**Measurement of outcome**	*Some concerns*	Measuring outcomes appropriated. Not specified whether outcome assessor not aware of the participants' assigned interventions. No apparent imbalances.
**Selection of reported result**	*Low risk*	Trial analyzed in accordance with a prespecified plan.
**Overall RoB2**	*Some concerns*	

Xu L (2017)

Entry	Judgment	Description
**Randomization process**	*Some concerns*	Block randomization using a computer-generated random allocation sequence reported. Allocation sequence concealed not described. No apparent imbalances.
**Deviations from intended interventions**	*Low risk*	Anesthesiologist delivering the interventions aware of participants' assigned intervention. Patients and outcome assessor not aware of the participants' assigned interventions. No apparent imbalances.
**Missing outcome data**	*Low risk*	Data about outcomes available for all participants. Result not biased by missing outcome data.
**Measurement of outcome**	*Low risk*	Measuring outcomes appropriated. Patients and outcome assessor not aware of the participants' assigned interventions. No apparent imbalances.
**Selection of reported result**	*Low risk*	Trial analyzed in accordance with a prespecified plan.
**Overall RoB2**	*Some concerns*	

Sayed NH (2021)

**Entry**	**Judgment**	**Description**
**Randomization process**	*Low risk*	Randomization using a computer-generated random allocation sequence reported. Allocation sequence concealed described. No apparent imbalances.
**Deviations from intended interventions**	*Some concerns*	Not specified whether anesthesiologist delivering the interventions and/or patients and/or outcome assessor are not aware of the participants' assigned interventions. No apparent imbalances.
**Missing outcome data**	*Low risk*	Data about outcomes available for all participants. Result not biased by missing outcome data.
**Measurement of outcome**	*Some concerns*	Measuring outcomes appropriated. Not specified whether patients and/or outcome assessor are not aware of the participants' assigned interventions. No apparent imbalances.
**Selection of reported result**	*Low risk*	Trial analyzed in accordance with a prespecified plan.
**Overall RoB2**	*Some concerns*	

Forest Plots Generated from Meta-Analysis for Various Endpoints Considered

Below is a series of forest plots corresponding to various endpoints considered in this study, including primary endpoints related to respiratory mechanics (Peak inspiratory pressure [P_{Peak}], plateau pressure [P_{Plat}], mean airway pressure [P_{Mean}], dynamic compliance [C_{Dyn}]),and secondary endpoints related to gas exchange (Arterial oxygen pressure [PaO_2], arterial carbon dioxide pressure [$PaCO_2$], pH), hemodynamics (Mean arterial pressure [MAP], heart rate [HR]), and inflammatory cytokines (Tumor necrosis factor-alpha [TNF-α]). These plots visually summarize the effect sizes derived from the data, with each plot representing the estimated effect for the specific outcome of interest.

In these forest plots, each study's effect size is presented, whether expressed as a mean difference (MD) for continuous outcomes or a Risk Ratio (RR) for binary outcomes. These are plotted against a central line of no effect, which represents a value of 0 for mean differences and 1 for RR, indicating no difference between the treatment and control groups.

The effect sizes are shown alongside their 95% confidence intervals (CIs), depicted as horizontal lines extending from each point estimate. The width of these intervals provides insight into the precision of the study's estimates, with narrower intervals suggesting higher precision.

The analysis was conducted using both fixed-effects and random-effects models within a frequentist framework to account for potential variability within and across studies. The random-effects model, preferred for the final analysis, assumes that the true treatment effects differ between studies, thus accounting for heterogeneity among study outcomes.

Comparisons presented in these forest plots were made between the treatment group (Inverse Ratio Ventilation) and the control group (Standard approach, Non-Inverse Ratio Ventilation). These visualizations provide a clear and detailed view of the relative effectiveness of the intervention across different variables, facilitating an informed understanding of its efficacy. By examining these plots, one can assess the statistical significance and potential impact of each intervention, contributing to a comprehensive evaluation of the outcomes considered in this study.

Forest Plots Generated from Meta-Analysis for Various Endpoints Considered before Pneumoperitoneum

P_{Peak} (cmH_2O)

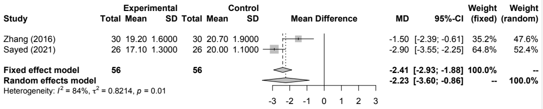

P_{Plat} (cmH_2O)

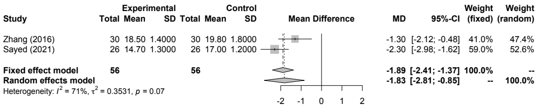

P_{Mean} (cmH_2O)

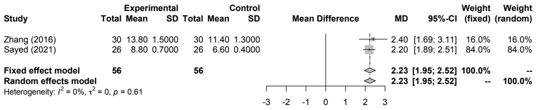

C_{Dyn} (mL/cmH_2O)

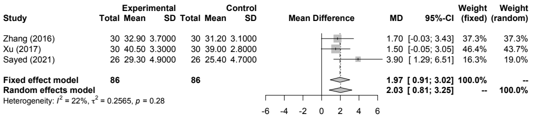

PaO_2 (mmHg)

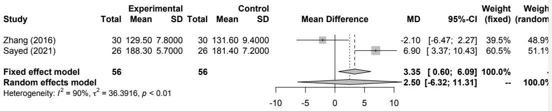

$PaCO_2$ (mmHg)

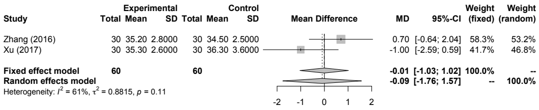

pH

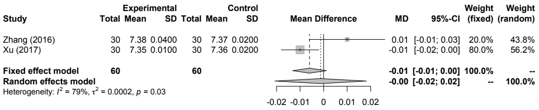

PAM (mmHg)

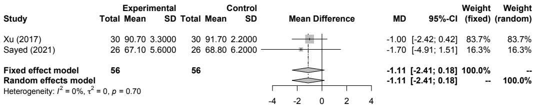

HR (beats/min)

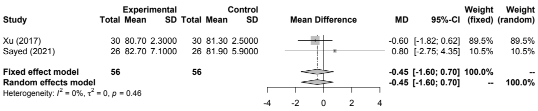

TNF-α (pg/mL)

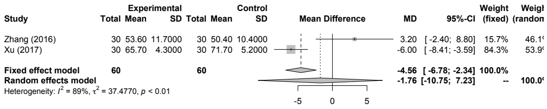

Forest Plots Generated from Meta-Analysis for Various Endpoints Considered during Pneumoperitoneum

P_{Peak} (cmH_2O)

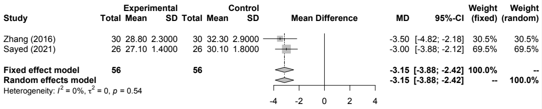

P_{Plat} (cmH_2O)

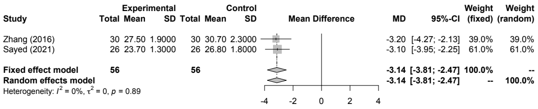

P_{Mean} (cmH_2O)

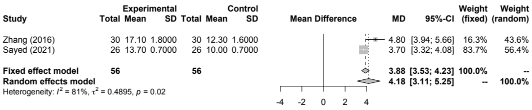

C_{Dyn} (mL/cmH_2O)

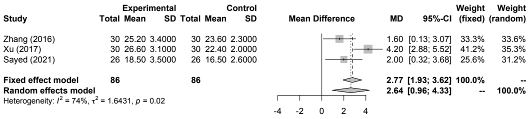

PaO_2 (mmHg)

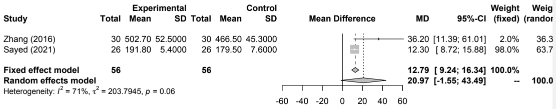

$PaCO_2$ (mmHg)

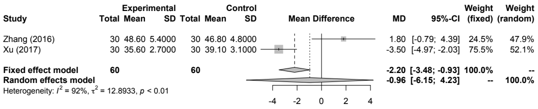

SUPPLEMENTARY MATERIAL (SM) 3 Forest plots

pH

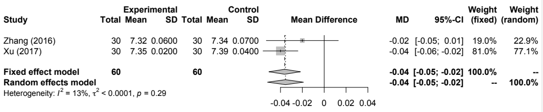

PAM (mmHg)

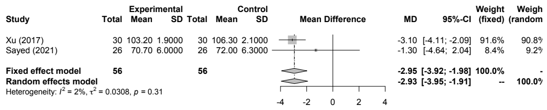

HR (beats/min)

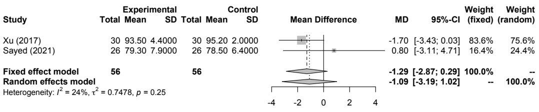

TNF-α (pg/mL)

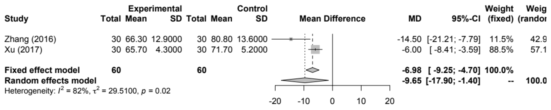

Postoperative Complications

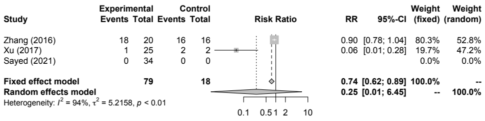

Funnel Plots Generated from Meta-Analysis for Various Endpoints Considered

Below is a series of funnel plots corresponding to various endpoints considered in this study, including primary endpoints related to respiratory mechanics (Peak inspiratory pressure [P_{Peak}], plateau pressure [P_{Plat}], mean airway pressure [P_{Mean}], dynamic compliance [C_{Dyn}]),and secondary endpoints related to gas exchange (Arterial oxygen pressure [PaO_2], arterial carbon dioxide pressure [$PaCO_2$], pH), hemodynamics (Mean arterial pressure [MAP], heart rate [HR]), and inflammatory cytokines (Tumor necrosis factor-alpha [TNF-α]). These plots are presented to assess the potential risk of publication bias and to evaluate the heterogeneity across the included studies.

In these funnel plots, the effect sizes from individual studies are plotted on the horizontal axis, centered around the comparison-specific effect. These effect sizes may be expressed as standardized mean differences or risk ratio, depending on the outcome being measured. The vertical axis displays the standard errors, with larger and more precise studies appearing at the top due to the reverse scaling of the standard errors.

The funnel shape is expected to be symmetrical if there is no publication bias or heterogeneity. The outer dashed lines define the triangular area where 95% of studies are expected to fall if there is no bias or heterogeneity. A vertical line in the plot indicates the overall estimate of the effect size based on the model. For continuous data, it is positioned at 0 (representing no effect), and for dichotomous data, this line is positioned at 1 (for Risk Ratio).

These funnel plots are used for visual inspection to assess the risk of publication bias in meta-analyses, offering a qualitative understanding of potential publication biases and study heterogeneity.

Funnel Plots Generated from Meta-Analysis for Various Endpoints Considered before Pneumoperitoneum

P_{Peak} (cmH_2O)

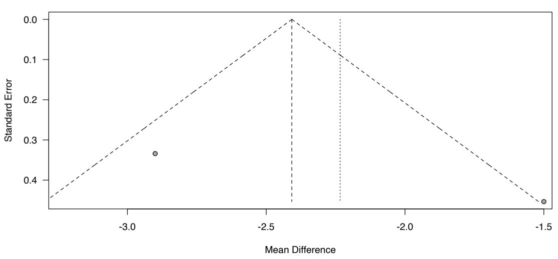

P_{Plat} (cmH_2O)

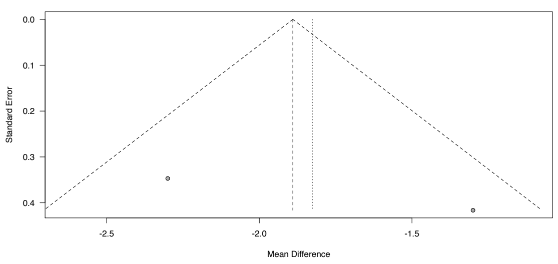

P_{Mean} (cmH_2O)

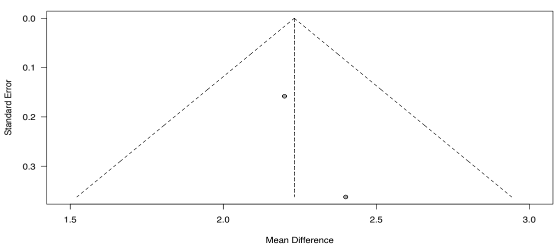

C_{Dyn} (mL/cmH_2O)

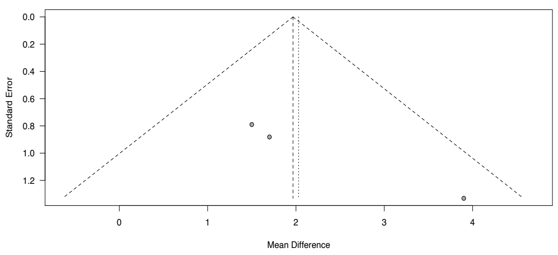

PaO_2 (mmHg)

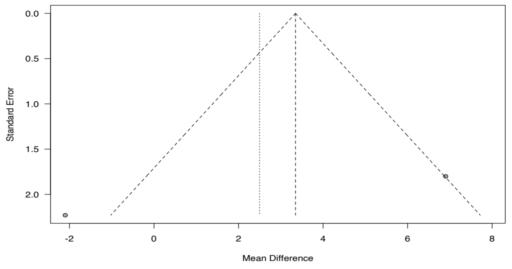

PaCO_2 (mmHg)

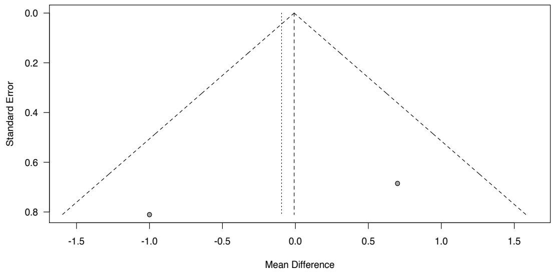

pH

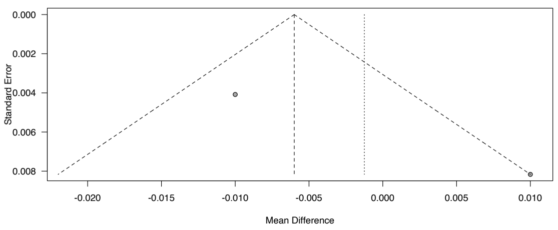

PAM (mmHg)

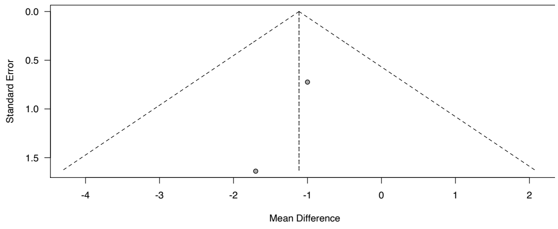

SUPPLEMENTARY MATERIAL (SM) 4 — Funnel plots

HR (beats/min)

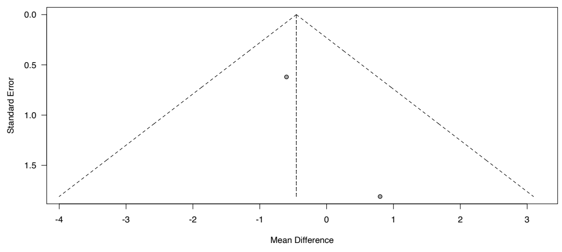

TNF-*α* (pg/mL)

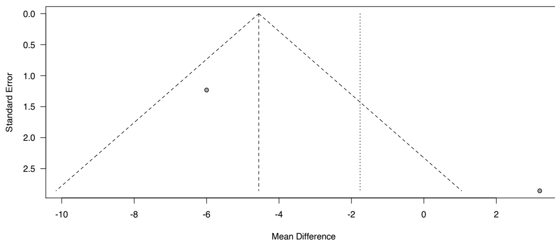

Funnel Plots Generated from Meta-Analysis for Various Endpoints Considered during Pneumoperitoneum

P_{Peak} (cmH_2O)

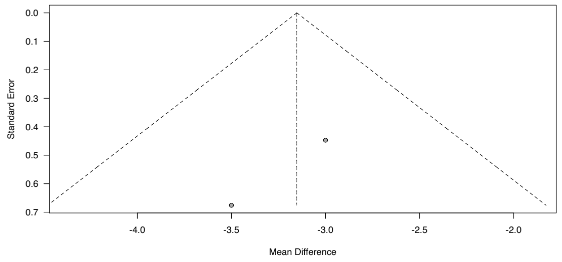

P_{Plat} (cmH_2O)

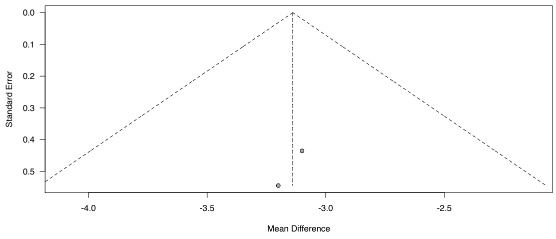

P_{Mean} (cmH_2O)

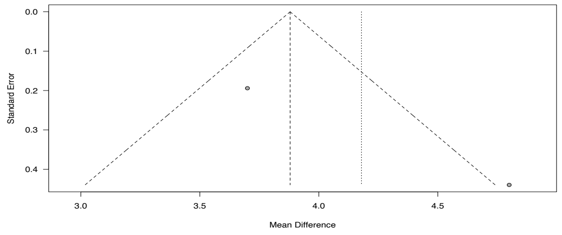

C_{Dyn} (mL/cmH_2O)

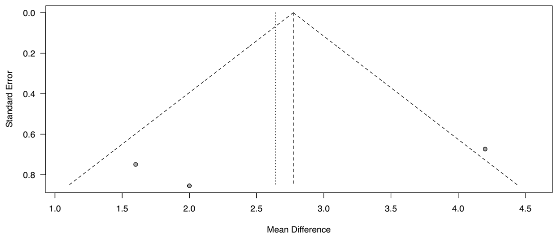

PaO_2 (mmHg)

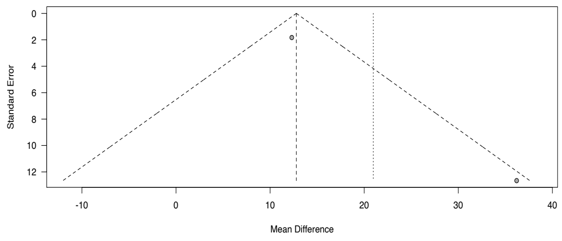

$PaCO_2$ (mmHg)

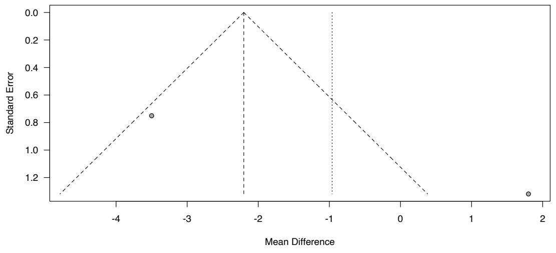

pH

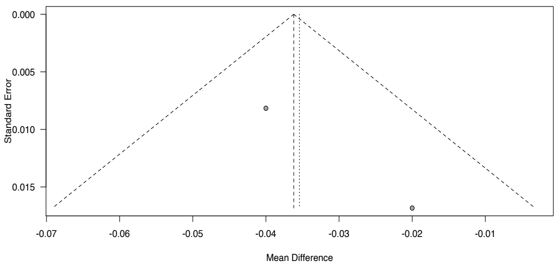

PAM (mmHg)

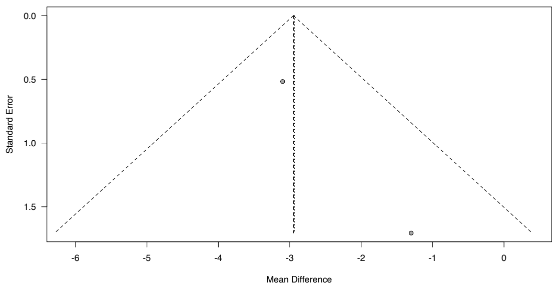

HR (beats/min)

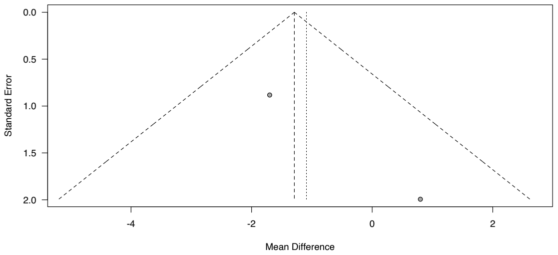

TNF-α (pg/mL)

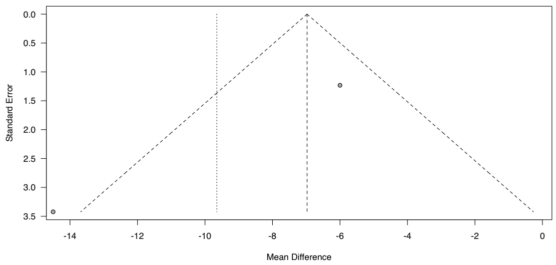

Postoperative Complications

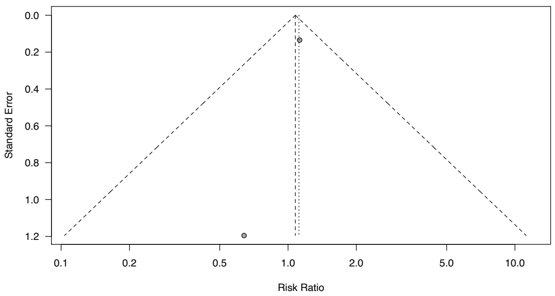

Supplement: Supplementary file 1 [file jcm-14-02063-s001.zip › jcm-3495271-supplementary.pdf]
